# Exogenous melatonin alleviates drought stress in cotton by enhancing root cortical activity and metabolic adaptation

**DOI:** 10.3389/fpls.2025.1625757

**Published:** 2025-07-02

**Authors:** Haitao Zhang, Congcong Guo, Hongchun Sun, Lingxiao Zhu, Ke Zhang, Yongjiang Zhang, Zhanbiao Wang, Cundong Li, Liantao Liu

**Affiliations:** ^1^ China Water-saving Agriculture, Ministry of Agriculture and Rural Affairs, Key Laboratory of Crop Growth Regulation of Hebei, College of Agronomy, Hebei Agricultural University, Baoding, China; ^2^ State Key Laboratory of Aridland Crop Science, Gansu Agricultural University, Lanzhou, China; ^3^ Institute of Western Agriculture, The Chinese Academy of Agricultural Sciences, Changji, China

**Keywords:** cotton, drought, melatonin, living cortical area, root metabolism, root absorption

## Abstract

**Introduction:**

The root cortex plays a critical role in water uptake and metabolic activity, directly influencing root functionality. However, despite melatonin’s known role in plant stress tolerance, its mechanisms in modulating root cortical anatomy and metabolic adaptation under drought remain unclear. This study examines the impact of exogenous melatonin on the root cortex of cotton under drought stress, focusing on its relationship with water uptake and drought resilience.

**Methods:**

Cotton plants (cv. Lumian 532) were subjected to drought stress (8% PEG 6000) with foliar application of melatonin (100 μmol/L) to evaluate its effects on root cortical integrity and water uptake.

**Results:**

The results demonstrated that melatonin application significantly increased living cortical area (LCA) and cortical thickness of roots under drought stress, and the effect was more obvious near the middle segment of the root (13 cm from the root tip). Melatonin also enhanced osmotic regulation, increased respiratory enzyme activity, and improved specific root length uptake rates for water and key nutrients. Furthermore, melatonin promoted root and above-ground growth, as evidenced by increases in root length, plant height, stem diameter, and leaf area. Notably, LCA positively correlated with osmotic substance accumulation, root respiration, and absorption capacity under drought conditions.

**Discussion:**

In conclusion, exogenous melatonin synergistically enhances drought resistance by maintaining cortical integrity, improving water absorption efficiency, and activating respiratory metabolism, thereby enhancing cotton growth and drought resistance. These findings underscore melatonin as a promising regulator for enhancing drought resistance.

## 1 Introduction

Cotton (*Gossypium hirsutum* L.) is a significant fiber and oilseed crop (Dong et al., 2008). Drought has emerged as a critical limiting factor affecting cotton production, severely impacting its growth, development, and yield (Chen and Dong, 2016). With the intensification of global climate change, the frequency and severity of drought events are expected to increase, further exacerbating water stress in cotton production, particularly in rain-fed agricultural areas (Ma et al., 2018). Therefore, enhancing the drought resistance of cotton is essential.

The structural and anatomical features of plant roots exhibit significant plasticity, which is crucial for adapting to drought stress. Studies have shown that moderate drought promotes root elongation, while severe drought inhibits normal root growth, leading to a decrease in indicators such as dry matter mass and root length (Mi et al., 2016). Guo et al. (2024a) found that smaller lateral root angles provide better access to deep soil moisture, which can help cotton to maintain a better physiological state and improve yield under drought conditions. The root cortical tissue, particularly in regions with strong absorptive functions, constitutes a substantial portion of the root system and serves as a vital site for the synthesis, storage, and secretion of nutrients and growth-regulating substances (Lynch, 2015). However, the cortex can consume over 50% of photosynthetic products to sustain survival, making it the primary region of the root system where photosynthetic resources are expended (Lambers et al., 2006). Therefore, changes in the root cortex under drought stress are critical for the adaptation of plant roots to drought conditions. Therefore, regulation of root growth and cortical area under drought stress is a major strategy for drought tolerance in plants.

The anatomical structure of the cortex is closely related to root metabolic consumption. Under soil stress conditions, the plant’s anatomical structure changes, adjusting the proportion of respiring and non-respiring root tissue to adapt to or alleviate adverse conditions (Schneider and Lynch, 2018). In short, drought stress diminishes plant productivity, disrupts the integrity of root cortical cells, reduces cellular metabolic activity, thereby inhibiting root respiration and decreasing root metabolic consumption (Chimungu et al., 2014). This indicates that the amount of living cells in the root cortex under drought stress is closely related to root respiratory metabolism. Structure influences function; the root cortex serves as a lateral transport pathway for water and nutrients into the vascular cylinder. A reduction in LCA of the roots will lead to decreased absorption capacity, thereby affecting water and nutrient uptake (Zhan and Lynch, 2015). Hu et al. (2014) found that as LCA of maize decreases, the radial transport of phosphates, calcium, and sulfates in maize roots also decreases. Therefore, an early reduction in the root cortical area can significantly regulate the source-sink relationship between the roots and aerial parts, resulting in stunted growth of the aerial portion, reduced photosynthetic capacity, and overall detriment to the plant.

Drought stress severely limits cotton production, and enhancing drought resistance is crucial for yield stability (Lobell et al., 2014). Exogenous plant growth regulators, such as melatonin, have shown promise in improving crop drought resistance (Guo et al., 2023). Melatonin is widely found in both plants and animals. In plants, it can be synthesized through both the classical pathway (tryptophan-5-hydroxytryptamine/serotonin) and an alternative pathway (5-methoxytryptamine) (Back et al., 2016). As a plant growth regulator, melatonin improves plant resistance to various biotic and abiotic stresses (Sun et al., 2021; Wang et al., 2022). Applying melatonin can promote root elongation in cotton and increase the number of lateral roots, thereby expanding the total root surface area and total root volume, resulting in a richer root system that fosters plant growth (Zhu et al., 2023). Under drought stress, excessive reactive oxygen species accumulation elevates malondialdehyde and hydrogen peroxide levels, causing membrane lipid peroxidation and cellular dysfunction (Møller et al., 2007). A study shows that exogenous melatonin enhances drought tolerance in soybean seedlings by regulating the antioxidant defense system to alleviate oxidative stress and reduce malondialdehyde accumulation (Zhao et al., 2025). Sharma et al. (2022) revealed that melatonin enhances drought tolerance in Arachis hypogaea by promoting jasmonic acid synthesis through modulating lipoxygenase expression and redox balance. Under drought stress, the application of exogenous melatonin significantly increases the accumulation of osmotic regulatory substances in the roots of hickory, providing protective effects and ensuring that the roots can maintain normal physiological functions in drought conditions (Wang et al., 2019). Thus, the root system is considered an important tissue through which melatonin enhances plant stress resistance.

The root cortex affects root activity and is a crucial site for reflecting the absorption function of the roots. Its functionality and physiological metabolism levels largely represent the overall functioning and metabolic status of the root system. Therefore, it is hypothesized that the regulatory effects of melatonin on the roots primarily target the cortical tissue. Previous studies have found that the application of melatonin under stress conditions significantly impacts the anatomical structure of plant roots. Research indicates that under salt stress, the cortical cells of cotton are the first to experience damage, exhibiting severe membrane rupture; however, melatonin can help maintain the structural integrity of the cortical cells, effectively alleviating damage caused by salt stress (Duan et al., 2022). Under drought stress, the application of melatonin significantly increases the size of the root epidermis, pith, cortex, secondary xylem, and vascular bundles in Eugenia uniflora, restoring them to their natural homeostasis (David et al., 2023). Hence, it is speculated that the exogenous application of melatonin would have a significant effect on the root cortex of cotton under drought stress.

The application of exogenous melatonin has been shown to mitigate the adverse effects of drought stress on plants and to influence the anatomical structure of the root system. However, few studies have investigated whether melatonin affects root metabolic processes and water and nutrient uptake by influencing cortical cells. Therefore, this study hypothesizes that under drought stress, exogenous melatonin can increase LCA of roots, prolong the functional integrity of the root cortex, promote cotton growth, and enhance drought resistance. The main objectives of this research are: (1) to elucidate the characteristics, patterns, and interrelationships of melatonin’s effects on LCA, osmotic regulatory substance content, and respiration rate in cotton roots under drought stress; (2) to reveal the impact of melatonin on the water and nutrient uptake rates of cotton roots under drought stress; (3) to explore the mechanisms by which changes in the activity of root cortical cells after the application of melatonin under drought stress enhance the drought resistance of cotton.

## 2 Materials and methods

### 2.1 Experimental design

The experiment was conducted from January to October 2024 in the artificial climate chamber at Hebei Agricultural University in Baoding, Hebei Province (38°85′N, 115°30′E). The cotton variety used in the study was “Lumian 532” Uniform and fully developed cotton seeds were selected, disinfected by soaking in 75% alcohol for 10 minutes and then rinsed with distilled water 5–8 times to remove all residual alcohol. After rinsing, the seeds were placed in distilled water and kept in a dark constant-temperature incubator at 25°C for 8 hours. The seeds were then spread out on a tray covered with a damp towel to germinate for 24 hours. Seeds with radicles approximately 1 cm long were transplanted onto a foam board with holes for hydroponic growth. Once the cotyledons fully expanded, seedlings with intact cotyledons and consistent growth were transplanted into hydroponic boxes (43 cm×30 cm×20 cm). Before use, the boxes were thoroughly disinfected with 50% carbendazim and then rinsed 2–3 times with clean water. In the hydroponic boxes, Hoagland nutrient solution was used, which was replaced every 3 days, and aeration was provided twice daily for 1 hour each time. The artificial climate chamber was set at 25°C/22°C (day/night), 600 μmol/m²/s light intensity, 14 hours photoperiod, and 60 ± 5% relative humidity.

When the seedlings developed three true leaves, uniform plants were selected for water and melatonin (MT) foliar spray treatments: CK (Well water, 0% PEG 6000), DS (Drought stress, 8% PEG 6000), CM (Well water and 100 μmol/L MT), and DM (Drought stress and 100 μmol/L MT). The concentration of 8% PEG 6000 (Guo et al., 2024b; Hu et al., 2017) and 100 μmol/L MT (Yang et al., 2023) were determined based on prior studies and validated in preliminary research by our group. During the experimental period, the CM and DM treatment groups received foliar applications of melatonin every 3 days, totaling 6 applications. In contrast, other treatments were sprayed with an equivalent volume of distilled water. Foliar spraying requires complete wetting of the front and back of the leaf until droplets are observed to begin forming and dripping from the leaf surface. Melatonin was applied at 8:00 PM under dark conditions to avoid photodegradation, as light exposure reduces its stability and efficacy.

### 2.2 Measurement of above-ground agronomic traits

On days 0, 6, 12, and 18 after treatment, plant height, stem diameter, and leaf area were measured, with five biological replicates for each measurement. Plant height was defined as the distance from the cotyledon node to the main shoot apex. Stem diameter was measured using a caliper at a point 1 cm above the cotyledon node. Leaf area was calculated using the length-width coefficient method (0.75).

### 2.3 Measurement of leaf relative water content

On days 0, 6, 12, and 18 after treatment, the relative water content of the third leaves from the main stem top was measured. Each measurement was repeated for three biological replicates. The relative water content was determined using the saturation-drying weighing method. In brief, leaves were immediately weighed (fresh weight), immersed in deionized water at 4°C for 8 to 12 hours (turgid weight), and oven-dried at 70°C for 72 hours (dry weight). The calculation formula is: Relative Water Content = (Fresh Weight - Dry Weight)/(Turgid Weight - Dry Weight) (Yang et al., 2022).

### 2.4 Measurement of chlorophyll fluorescence parameters

On days 0, 6, 12, and 18 after treatment, chlorophyll fluorescence parameters of main stem functional leaves in cotton plants were measured using a PAM-2500 portable chlorophyll fluorometer (Walz, Germany), with five biological replicates for each measurement. Prior to measurements, target leaves were subjected to 30-minute dark adaptation using leaf clips, after which dynamic changes in PSII maximum photochemical efficiency(Fv/Fm) and PSII actual photochemical quantum yield (ΦPSII) were recorded across treatments.

### 2.5 Measurement of root traits

On days 0, 6, 12, and 18 after treatment, total root length, total root surface area, and total root volume were measured, with three biological replicates for each measurement. The roots were placed without overlapping in a transparent acrylic box with a water depth of 1 cm for scanning (Epson V700; Epson, Suwa, Japan) to obtain root images. The total root length, total root surface area, and total root volume were analyzed using WinRHIZO software (WinRHIZO REG 2009, Canada).

### 2.6 Measurement of biomass

On days 0, 6, 12, and 18 after treatment, root and above-ground biomass were measured, with three biological replicates for each measurement. The roots and above-ground parts of the seedlings were separated and placed in bags. They were then heated at 105°C for 30 minutes to inactivate enzymes and dried at 80°C until reaching a constant weight to determine the biomass of the roots and aerial parts.

### 2.7 Measurement of water and nutrient uptake rates

On day 18 after treatment, the specific root length uptake rates of water, nitrate, ammonium, and phosphate in the seedlings were measured, with three biological replicates and three technical replicates for each measurement. The seedlings were preconditioned by transferring them to nutrient solution lacking macroelements for 48 hours prior to measurement. Twelve conical flasks containing 260 ml of normal nutrient solution were prepared, and 2 ml of the solution was collected as a sample before transferring the seedlings. After the seedlings were transferred to the conical flasks for 4 hours, the remaining volume of the nutrient solution in each flask was measured. The water uptake rate was calculated based on the difference in nutrient solution volume before and after uptake. Additionally, 2 ml samples of the remaining nutrient solution were collected and stored at -20°C. Root images were captured at the end of sampling for total root length analysis. A continuous flow chemical analyzer (SmartChem 600, AMS Alliance, France) was used to determine the concentrations of nitrate, ammonium, and phosphate in the water samples, and the nutrient uptake rates were calculated based on the difference in nutrient concentration before and after uptake (Roy et al., 2021).

### 2.8 Analysis of root cortex anatomy

**Figure 1:**
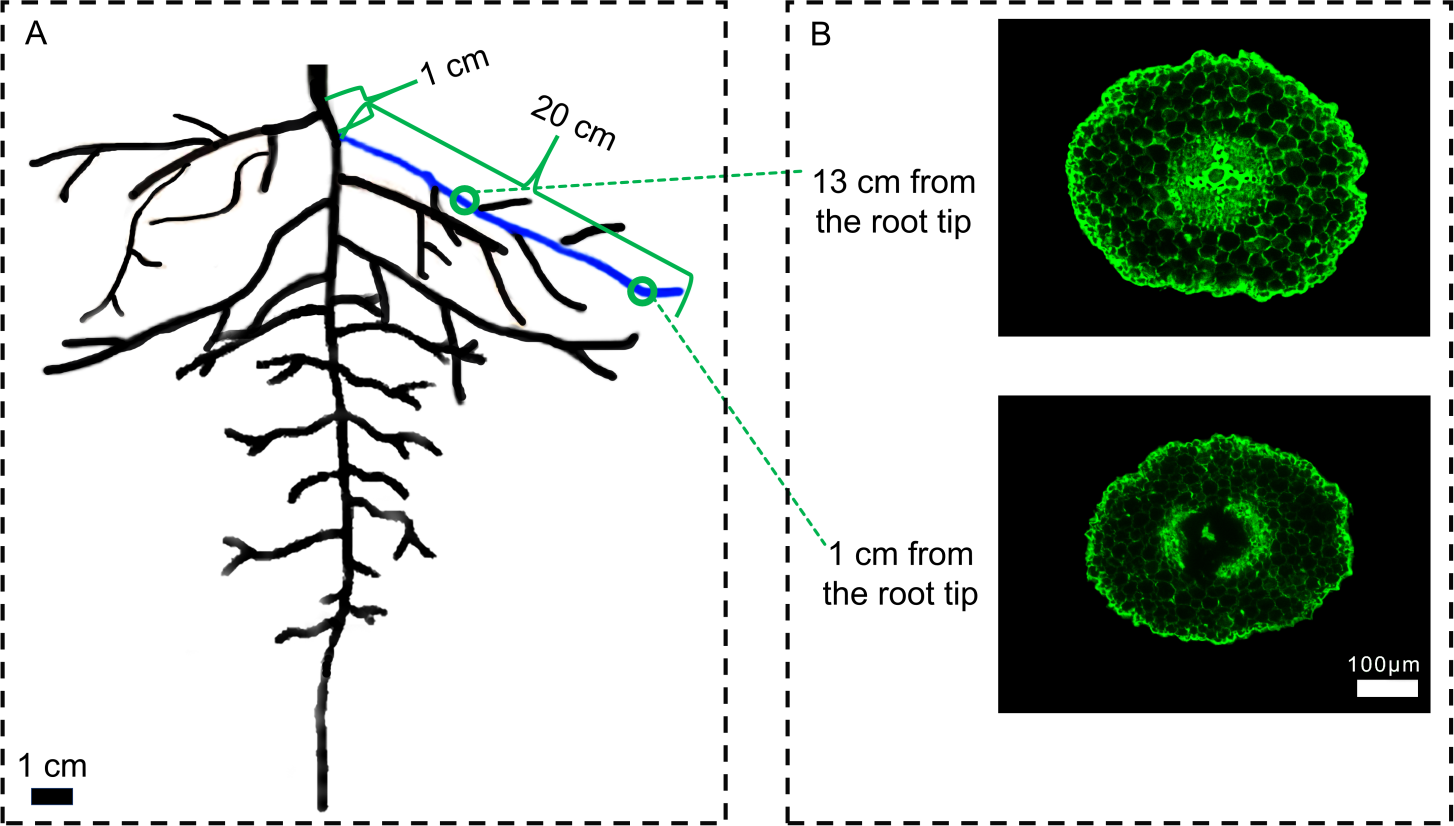
Schematic diagram of sampling for root cortex anatomy. **(A)** Schematic diagram of the cotton root, **(B)** Cross-sectional view of a cotton root segment. 1 cm represents the distance from the lateral root growth point to the base of the main root. 20 cm is the length of the sampled lateral root. The blue part indicates the selected lateral root. The green rings mark the positions for taking 0.5 cm root segments at different distances from the root tip. Scale bar **(A)** 1 cm. Scale bar **(B)** 100 μm.

On days 0, 6, 12, and 18 after treatment, the root cortex cells were examined. Lateral roots were selected at a position 1 cm from the base of the main root, with a lateral root length of approximately 20 cm. Root segments were taken from the lateral roots at 1 cm and 13 cm from the root tip (0.5 cm segments), with three biological replicates and three technical replicates for each measurement (**Figure 1**). The 1 cm position corresponds to the root segment that has just entered the maturation zone in cotton, where cortical cells are newly differentiated and structurally more intact compared to older segments. In contrast, the 13 cm position is located in the middle part of the root and represents a fully mature root segment undergoing senescence. The collected root segments were immediately placed in FAA fixative and fixed at 4°C for 24 hours. Using the paraffin sectioning technique, the roots were subjected to dehydration, clarification, embedding, and sectioning to produce anatomical slides. After staining with acridine orange (Henry and Deacon, 1981), images of the sections were captured using a fluorescence microscope (Axio Imager M2, Zeiss, Germany). The root cross-sectional area, stele area, and cortical thickness were measured using ImageJ software, and LCA of roots was calculated as (root cross-sectional area - stele area) (Jaramillo et al., 2013).

### 2.9 Measurement of osmotic regulatory substances

On days 0, 6, 12, and 18 after treatment, the content of soluble sugar and soluble protein were measured, with three biological replicates and three technical replicates for each measurement. In brief, 0.2 g of plant roots was weighed, rapidly frozen in liquid nitrogen, and stored at -80°C. The soluble sugar content was determined by anthrone colorimetry, and the soluble protein content was determined by Caulmers Brilliant Blue method, both of which were determined using the kits from Nanjing Jianjian Bioengineering Research Institute (the numbering information was A145-1–1 and A045–2 in order).

### 2.10 Measurement of root respiration rate and activity of respiratory metabolism enzymes

On days 0, 6, 12, and 18 after treatment, the root respiration rate and the activity of respiratory metabolism enzymes in the roots were measured, with three biological replicates and three technical replicates for each measurement. In brief, a Li-840A CO_2_ analyzer was used for measurement. Clean roots were quickly dried of surface moisture using absorbent paper and immediately placed into a custom-made 18 ml respiration chamber. The gas circuit was maintained as closed throughout the measurement process, and measurements were completed when the curve stabilized. The roots were then collected for total root length measurement, and the specific root length respiration rate was calculated. The activities of phosphofructokinase, glucose-6-phosphate dehydrogenase, and malate dehydrogenase in roots were determined using the kits provided by Abbkine (Scientific Co., Ltd, Wuhan, China) (the numbering information was KTB1124, KTB1011 and KTB3021 in order). Sampling was conducted according to “Measurement of osmotic regulatory substances”, and the determination was carried out in accordance with the kit instructions.

### 2.11 Statistical analysis

Data recording and organization were performed using Microsoft Excel 2010 (Microsoft Corporation, Redmond, WA, USA). One-way ANOVA was conducted using IBM SPSS Statistics 27.0 (IBM Corp, Armonk, NY, USA), and the Duncan method was used for multiple comparisons and significance tests, with a significance level of *p* < 0.05. Graphs were created using GraphPad Prism 9.0 (GraphPad Software, Inc., San Diego, CA, USA), and correlation analysis charts were plotted using Origin 2021 (OriginLab Corporation, Northampton, MA, USA).

## 3 Results

### 3.1 Effects of exogenous melatonin on the above-ground growth of cotton under drought stress

**Figure 2:**
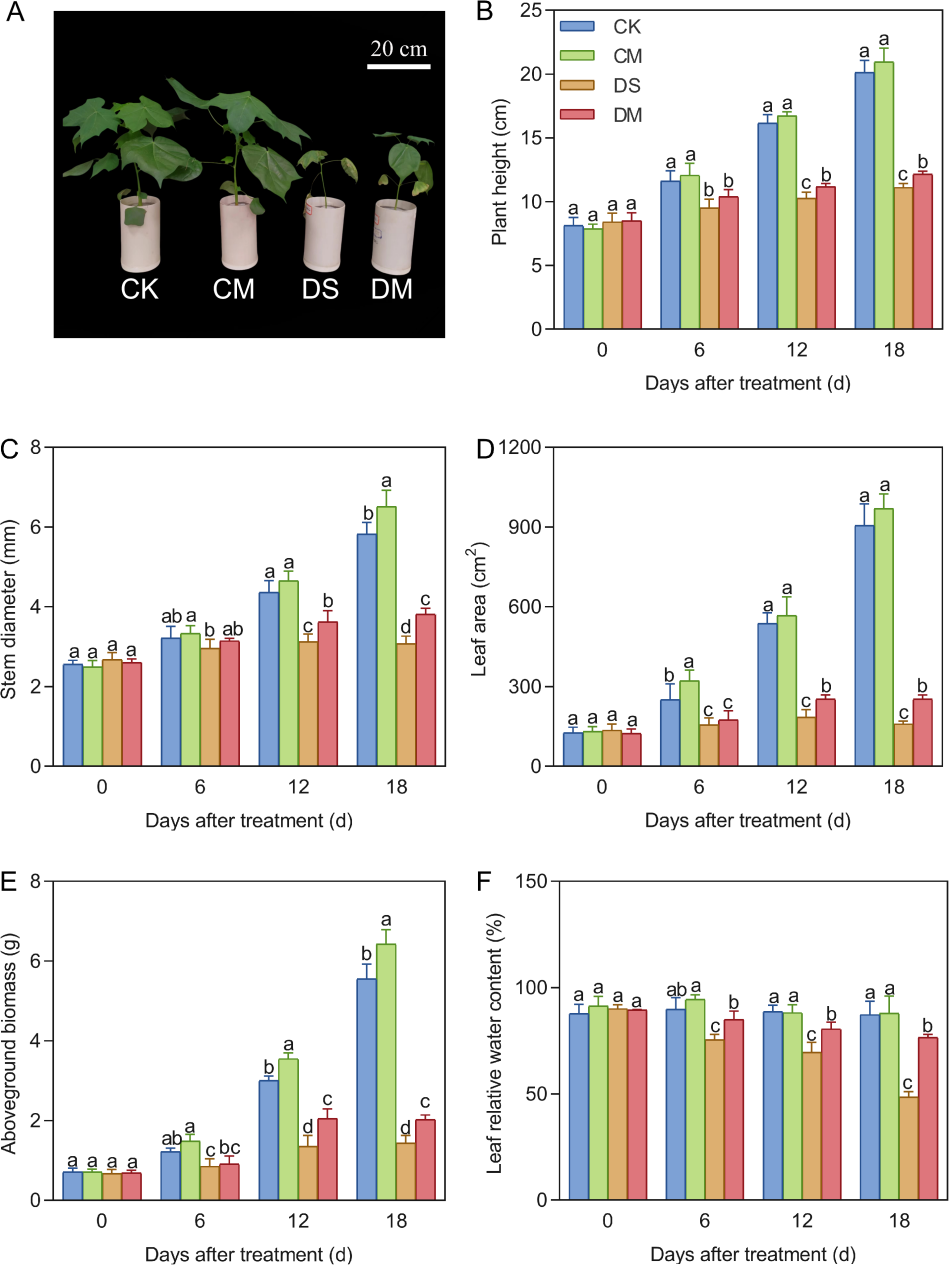
Effects of different treatments on above-ground indicators. **(A)** Phenotype of cotton 18 days after treatment, **(B)** Plant height, **(C)** Stem diameter, **(D)** Leaf area, **(E)** Above-ground biomass, and **(F)** Leaf relative water content. CK, well water; CM, well water and melatonin; DS, drought stress; DM, drought stress and melatonin. Different lowercase letters indicate significant differences among different treatments at the same time period (*p* < 0.05). **(B-D)** Values are means ± SE (n=5), **(E, F)** Values are means ± SE (n=3). Scale bar: 20 cm.

Melatonin significantly improved plant height, stem diameter, leaf area, above-ground biomass, and leaf water content under drought stress (**Figure 2**). Compared with the control, after 18 days of drought stress treatment, the plant height, stem diameter, leaf area, above-ground biomass, and leaf relative water content decreased by 44.73%, 47.20%, 82.39%, 74.06%, and 44.40%, respectively (*p* < 0.05). However, exogenous application of melatonin could promote the growth and development of cotton, especially under drought stress conditions. Specifically, compared with the well water treatment, the stem diameter and above-ground biomass of the well water and melatonin treatment increased significantly by 10.53% and 15.72%, respectively (*p* < 0.05); Meanwhile, compared with the drought stress treatment, the plant height, stem diameter, leaf area, above-ground biomass, and leaf relative water content of the drought stress and melatonin treatment increased significantly by 9.35%, 24.15%, 58.49%, 40.63%, and 57.56% respectively (*p* < 0.05).

**Figure 3:**
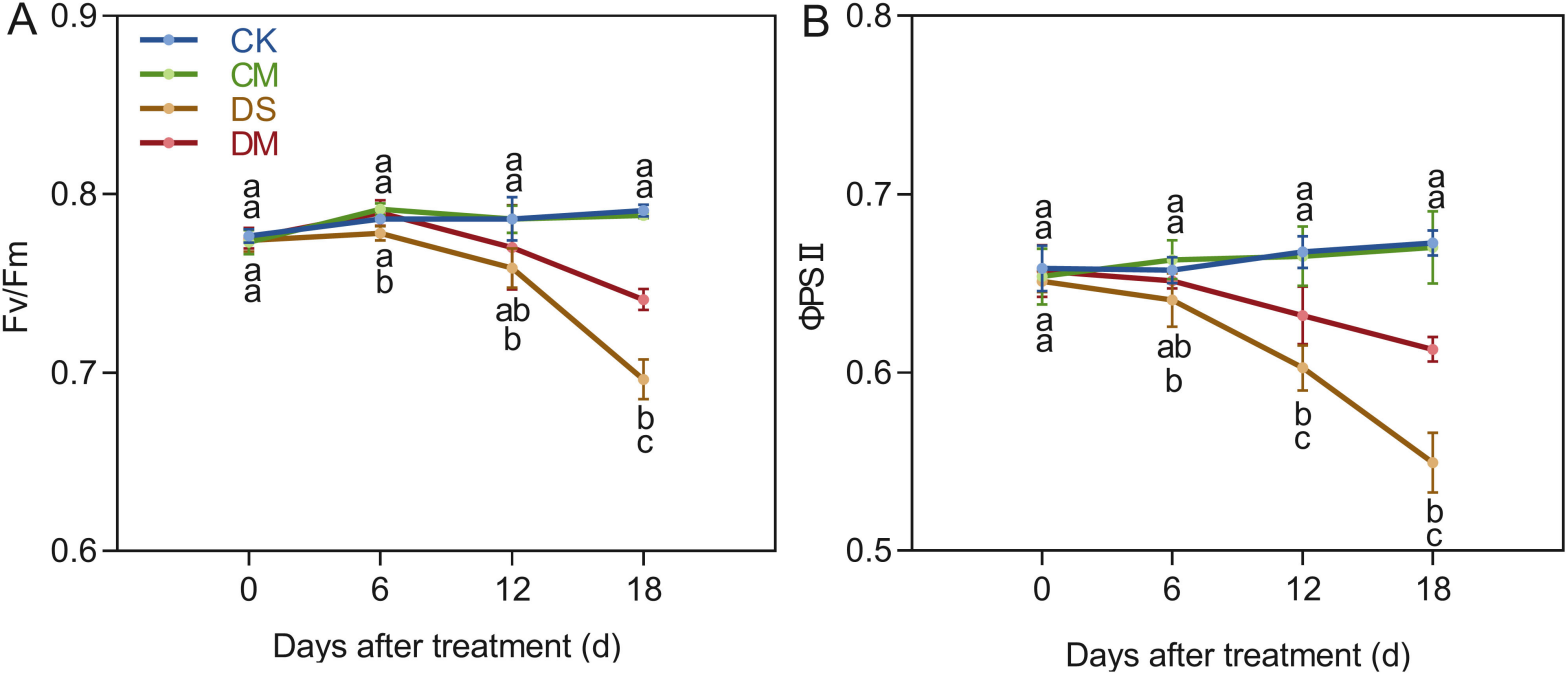
Effects of different treatments on chlorophyll fluorescence parameters. **(A)** PSII maximum photochemical efficiency(Fv/Fm) and **(B)** PSII actual photochemical quantum yield (ΦPSII). CK, well water; CM, well water and melatonin; DS, drought stress; DM, drought stress and melatonin. Different lowercase letters indicate significant differences among different treatments at the same time period (*p* < 0.05). Values are means ± SE (n=5).

Melatonin significantly improved chlorophyll fluorescence parameters under drought stress (**Figure 3**). With cotton growth, Fv/Fm and ΦPSII remained basically stable under well water treatment, while both gradually decreased under drought stress. Compared with the control, after 18 days of drought stress treatment, Fv/Fm and ΦPSII decreased by 11.96% and 18.32%, respectively (*p* < 0.05). However, exogenous application of melatonin significantly increased Fv/Fm and ΦPSII under drought stress. Specifically, compared with the drought stress treatment, the Fv/Fm and ΦPSII of the drought stress and melatonin treatment increased significantly by 6.43% and 11.58% respectively (*p* < 0.05). In summary, drought stress significantly inhibited the activity of photosystem II in cotton, while exogenous melatonin effectively alleviated the damage of drought to photosynthetic functions and improved the stability of light energy utilization under stress by maintaining light energy conversion efficiency.

### 3.2 Effects of exogenous melatonin on the growth and development of cotton roots under drought stress

**Figure 4:**
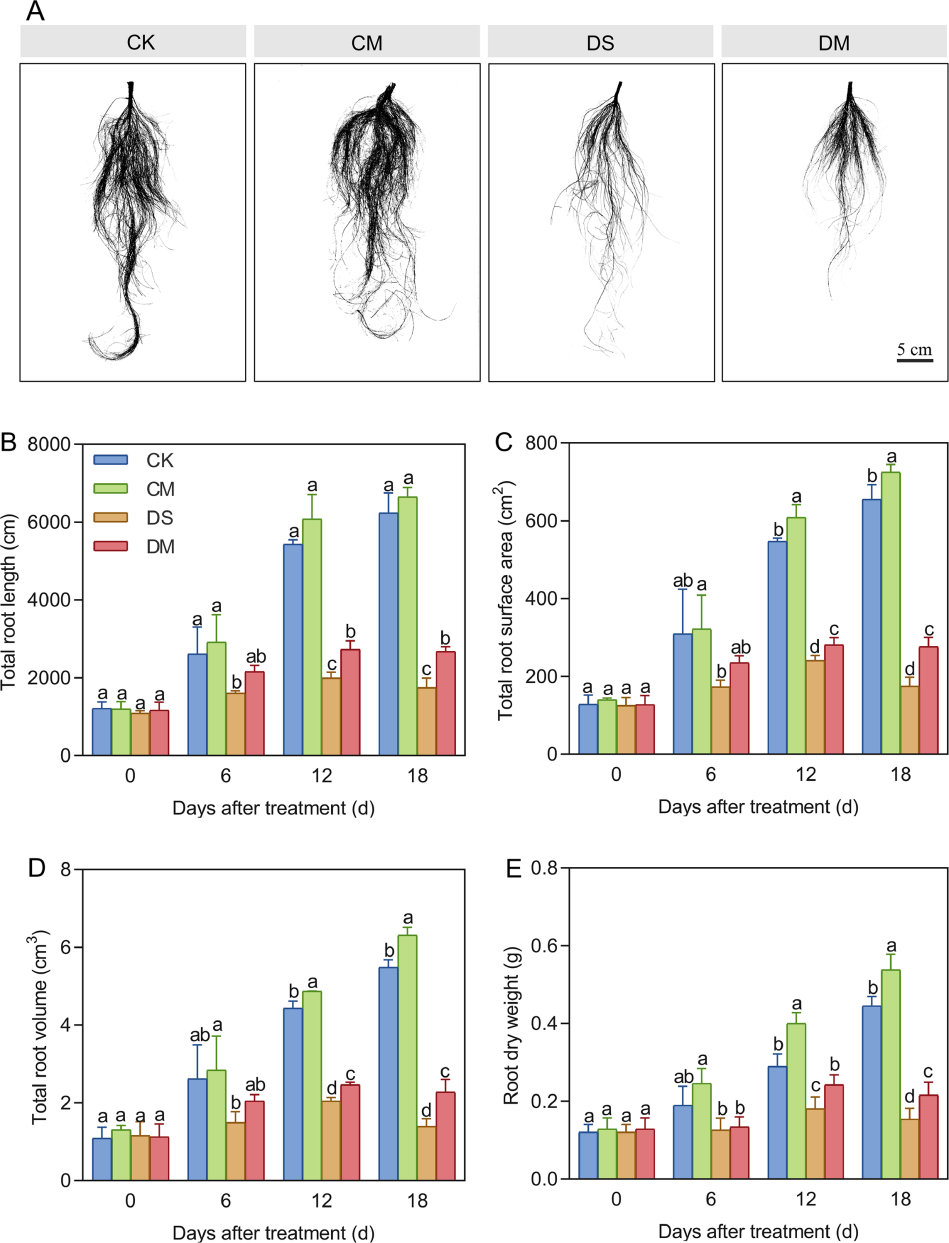
Effects of different treatments on root morphological indices. **(A)** Root phenotype of cotton 18 days after treatment, **(B)** Total root length, **(C)** Total root surface area, **(D)** Total root volume, and **(E)** Root dry weight. CK, well water; CM, well water and melatonin; DS, drought stress; DM, drought stress and melatonin. Different lowercase letters indicate significant differences among different treatments at the same time period (*p* < 0.05). Values are means ± SE (n=3). Scale bar: 5 cm.

Melatonin enhanced total root length, total root surface area, total root volume, and root dry weight under drought stress (**Figure 4**). Compared with the control, after 18 days of drought stress treatment, the total root length, total root surface area, total root volume, and root dry weight decreased by 71.96%, 73.36%, 74.58%, and 65.45%, respectively (*p* < 0.05). The root/shoot ratio of the drought stress treatment was significantly increased by 39.37% and 32.57% compared with the drought stress and melatonin treatment on days 12 and 18 after treatment, respectively(*p* < 0.05) (**Supplementary Figure S1**). However, exogenous application of melatonin could promote the growth and development of roots, especially under drought-stress conditions. Specifically, compared with the well water treatment, the total root surface area, total root volume, and root dry weight of the well water and melatonin treatment increased significantly by 10.71%, 15.01%, and 20.72%, respectively (*p* < 0.05); Meanwhile, compared with the drought stress treatment, the total root length, total root surface area, total root volume, and root dry weight of the drought stress and melatonin treatment increased significantly by 52.90%, 58.36%, 63.56%, and 40.26%, respectively (*p* < 0.05). In conclusion, exogenous application of melatonin can alleviate the growth inhibition of cotton roots caused by drought stress and play a significantly promoting role in the growth of cotton roots.

### 3.3 Effects of exogenous melatonin on the uptake rates of water and nutrients in cotton roots under drought stress

**Figure 5:**
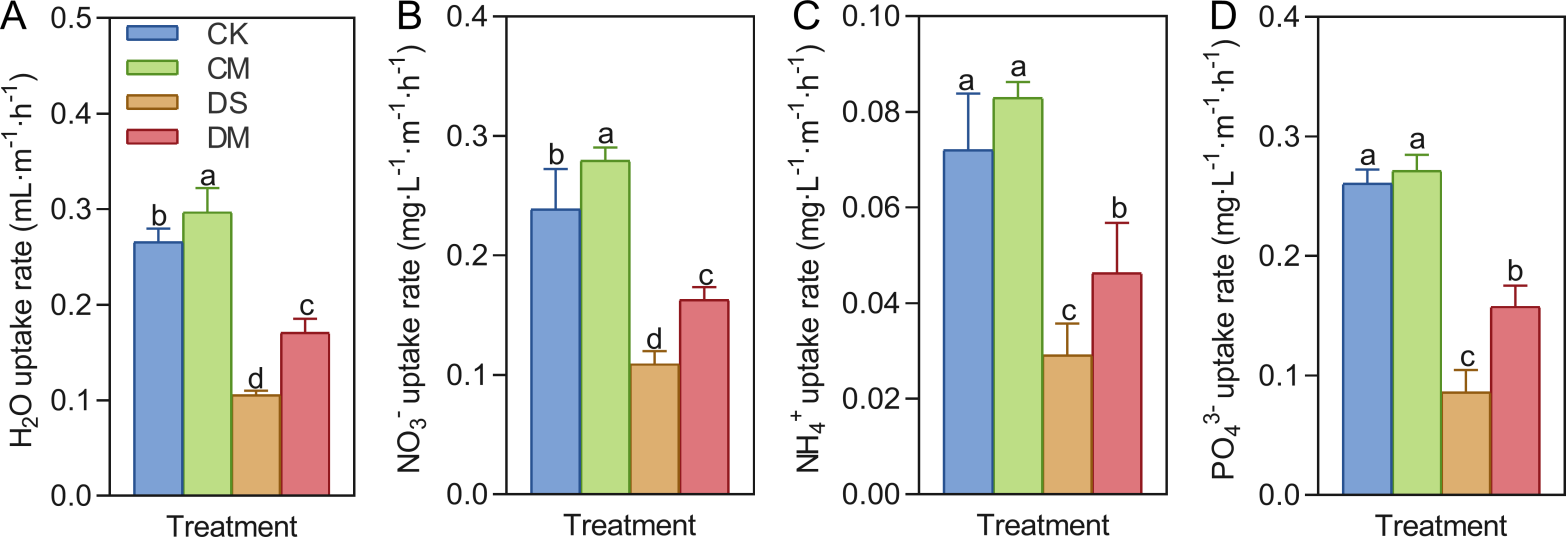
Effects of different treatments on the specific root length absorption rate 18 days after treatment. **(A)** Water uptake rate, **(B)** Nitrate uptake rate, **(C)** Ammonium salt uptake rate, and **(D)** Phosphate uptake rate. CK, well water; CM, well water and melatonin; DS, drought stress; DM, drought stress and melatonin. Different lowercase letters indicate significant differences among different treatments in the same period (*p* < 0.05). Values are means ± SE (n=3).

Melatonin significantly promoted the uptake rates of water, nitrate, ammonium, and phosphate in cotton roots under drought-stress treatment (**Figure 5**). Compared with the control, after 18 days of drought stress treatment, the specific root length uptake rates of water, nitrate, ammonium, and phosphate decreased by 60.25%, 54.40%, 59.60%, and 67.01%, respectively (*p* < 0.05). However, exogenous application of melatonin could enhance the absorption capacity of roots, especially under drought-stress conditions. Specifically, compared with the well water treatment, the specific root length uptake rates of water and nitrate in the well water and melatonin treatment increased significantly by 11.85% and 16.89%, respectively (*p* < 0.05); Meanwhile, compared with the drought stress treatment, the specific root length uptake rates of water, nitrate, ammonium, and phosphate of the drought stress and melatonin treatment increased significantly by 61.97%, 49.52%, 59.21%, and 83.26%, respectively (*p* < 0.05).

### 3.4 Effects of exogenous melatonin on the root cortex of cotton seedlings under drought stress

**Figure 6:**
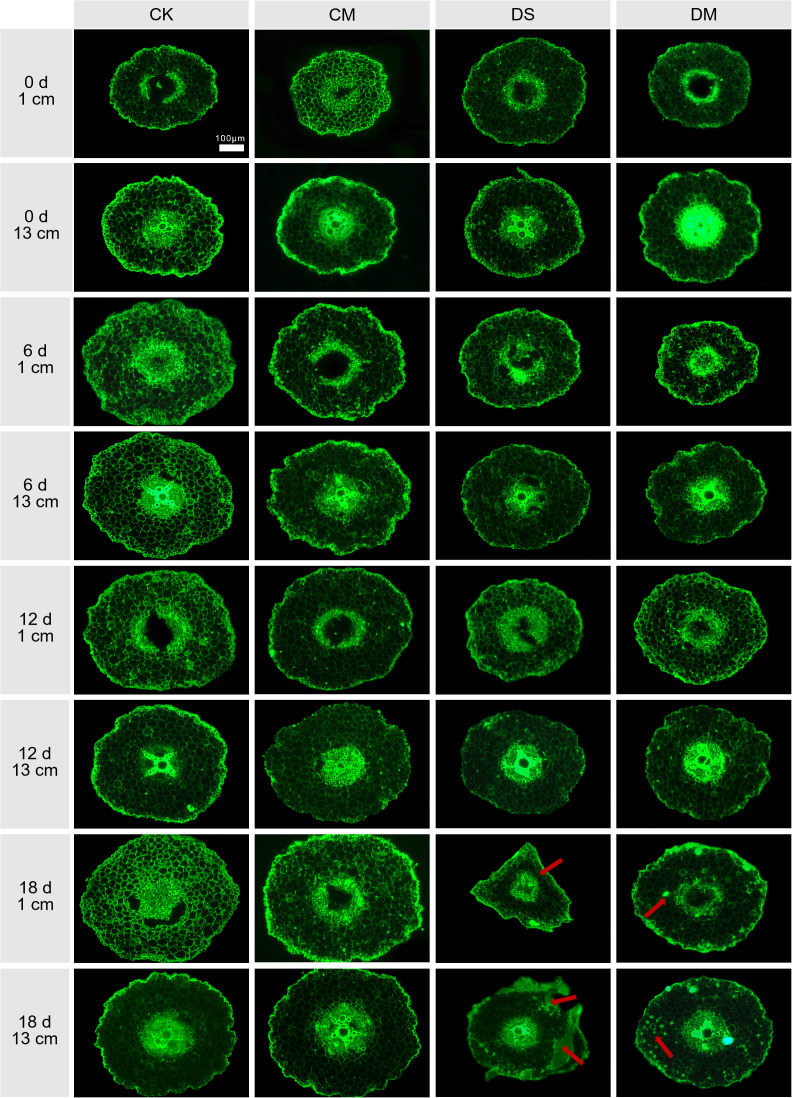
Effects of different treatments on the root cross-sections at 1 cm and 13 cm from the root tip at 0 d, 6 d, 12 d and 18 d after treatment. CK, well water; CM, well water and melatonin; DS, drought stress; DM, drought stress and melatonin. The red arrow indicates the cortical damage area. Scale bar: 100 μm.

**Figure 7:**
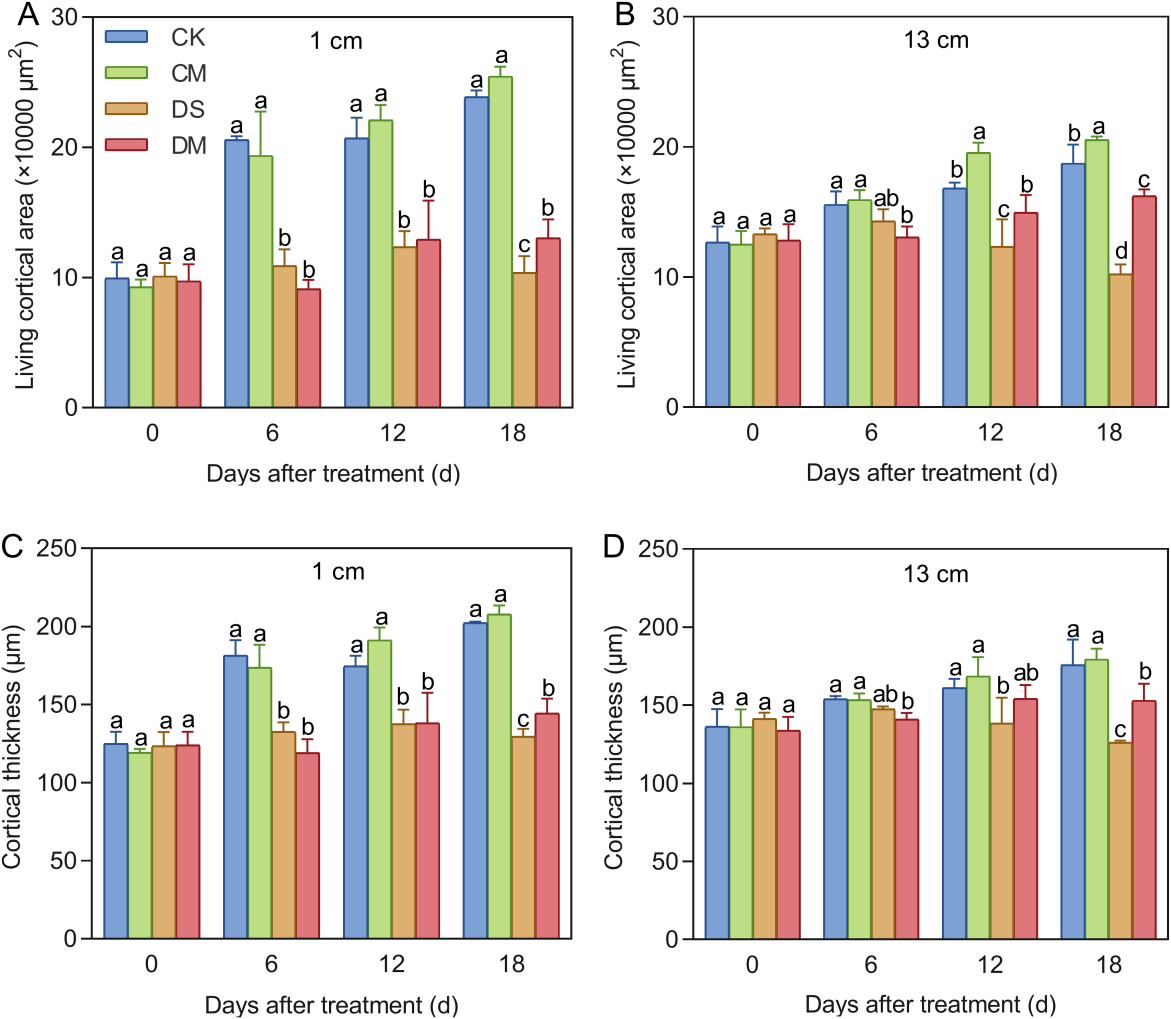
Effects of different treatments on root cortex cells. **(A)** LCA at 1 cm from the root tip, **(B)** LCA at 13 cm from the root tip, **(C)** Cortical thickness at 1 cm from the root tip, and **(D)** Cortical thickness at 13 cm from the root tip. CK, well water; CM, well water and melatonin; DS, drought stress; DM, drought stress and melatonin. Different lowercase letters indicate significant differences among different treatments at the same time period (*p* < 0.05). Values are means ± SE (n=3).

Melatonin significantly promoted LCA and cortical thickness of cotton roots under drought-stress treatment (**Figures 6**, **7**). The data showed that at a position 1 cm from the root tip, Compared with the control, after 18 days of drought stress treatment, LCA and cortical thickness of the roots decreased by 56.55% and 36.00%, respectively (*p* < 0.05) (**Figures 7A, C**). However, exogenous application of melatonin could promote LCA and cortical thickness of cotton roots under drought stress. Specifically, LCA and cortical thickness in the drought stress and melatonin treatment were significantly increased by 25.72% and 11.46% compared with the drought stress treatment (*p* < 0.05) (**Figure 7A, C**). Meanwhile, at a position 13 cm from the root tip, significant differences began to appear on the 12th day. Compared with the well water, after 18 days of drought stress treatment, LCA and cortical thickness of the roots decreased by 45.50% and 28.19%, respectively (*p* < 0.05), while LCA of the roots in the well water and melatonin treatment was significantly increased by 9.69% (*p* < 0.05). At the same time, compared with the drought stress treatment, LCA and cortical thickness of the roots in the drought stress and melatonin treatment were significantly increased by 59.00% and 21.24%, respectively (*p* < 0.05) (**Figures 7B, D**). In conclusion, exogenous application of melatonin can maintain the activity of root cortex cells in cotton under drought stress and improve root adaptability.

### 3.5 Effects of exogenous melatonin on the contents of osmotic regulatory substances in the roots of cotton seedlings under drought stress

**Figure 8:**
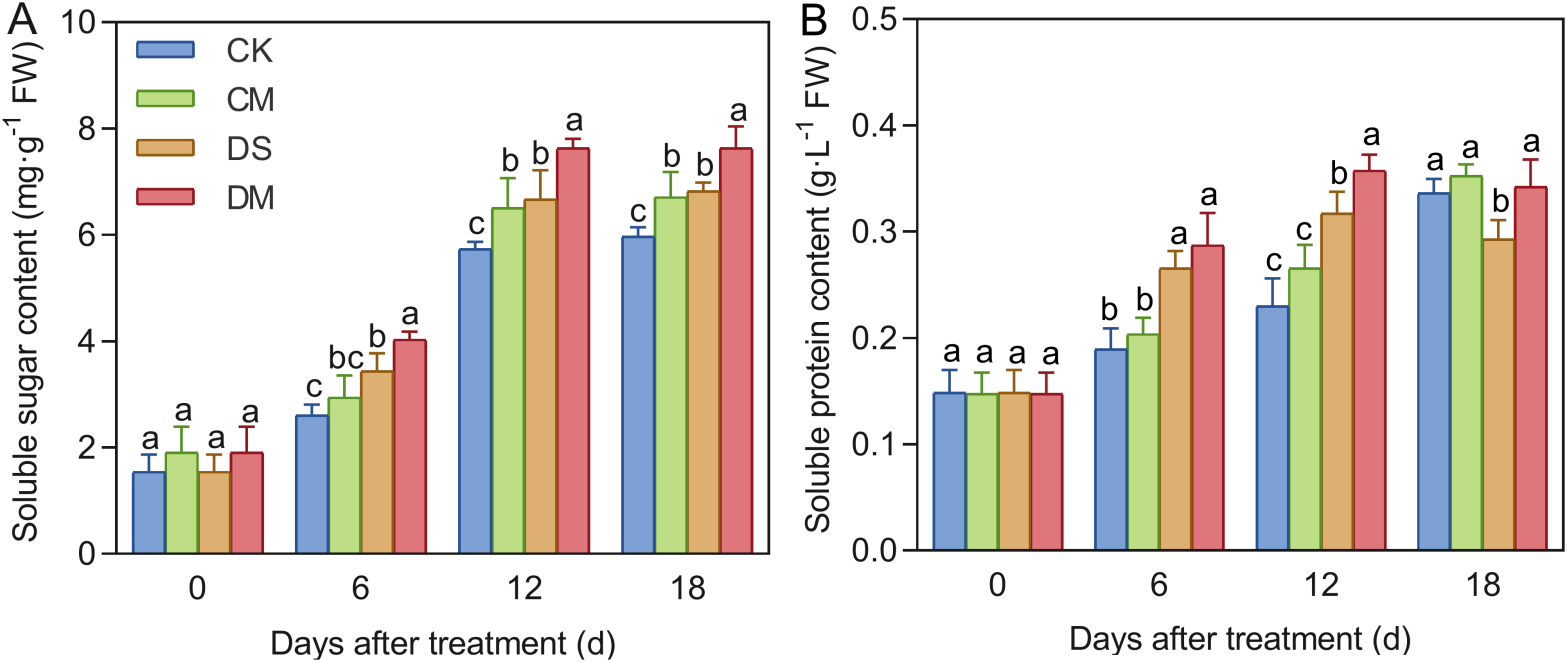
Effects of different treatments on osmotic regulatory substances in roots. **(A)** Soluble sugar content, and **(B)** Soluble protein content. CK, well water; CM, well water and melatonin; DS, drought stress; DM, drought stress and melatonin. Different lowercase letters indicate significant differences among different treatments at the same time period (*p* < 0.05). Values are means ± SE (n=3).

Melatonin significantly promoted the accumulation of soluble sugar and soluble protein in cotton seedlings under drought-stress treatment (**Figure 8**). Compared with the control, after 18 days of drought stress treatment, the content of soluble sugars increased by 14.31% (*p* < 0.05), while the content of soluble protein decreased significantly by 12.88% (*p* < 0.05). However, exogenous application of melatonin could effectively increase the osmotic regulatory substances. Specifically, compared with the well water treatment, the content of soluble sugars in the well water and melatonin treatment increased significantly by 12.37% (*p* < 0.05); Meanwhile, compared with the drought stress treatment, the content of soluble sugar and soluble protein of the drought stress and melatonin treatment increased significantly by 11.90% and 16.75%, respectively (*p* < 0.05). In conclusion, exogenous application of melatonin plays a significantly promoting role in increasing the content of soluble sugar and soluble protein under drought stress, and can effectively alleviate the damage to roots caused by drought stress.

### 3.6 Effects of exogenous melatonin on root metabolism of cotton seedlings under drought stress

**Figure 9:**
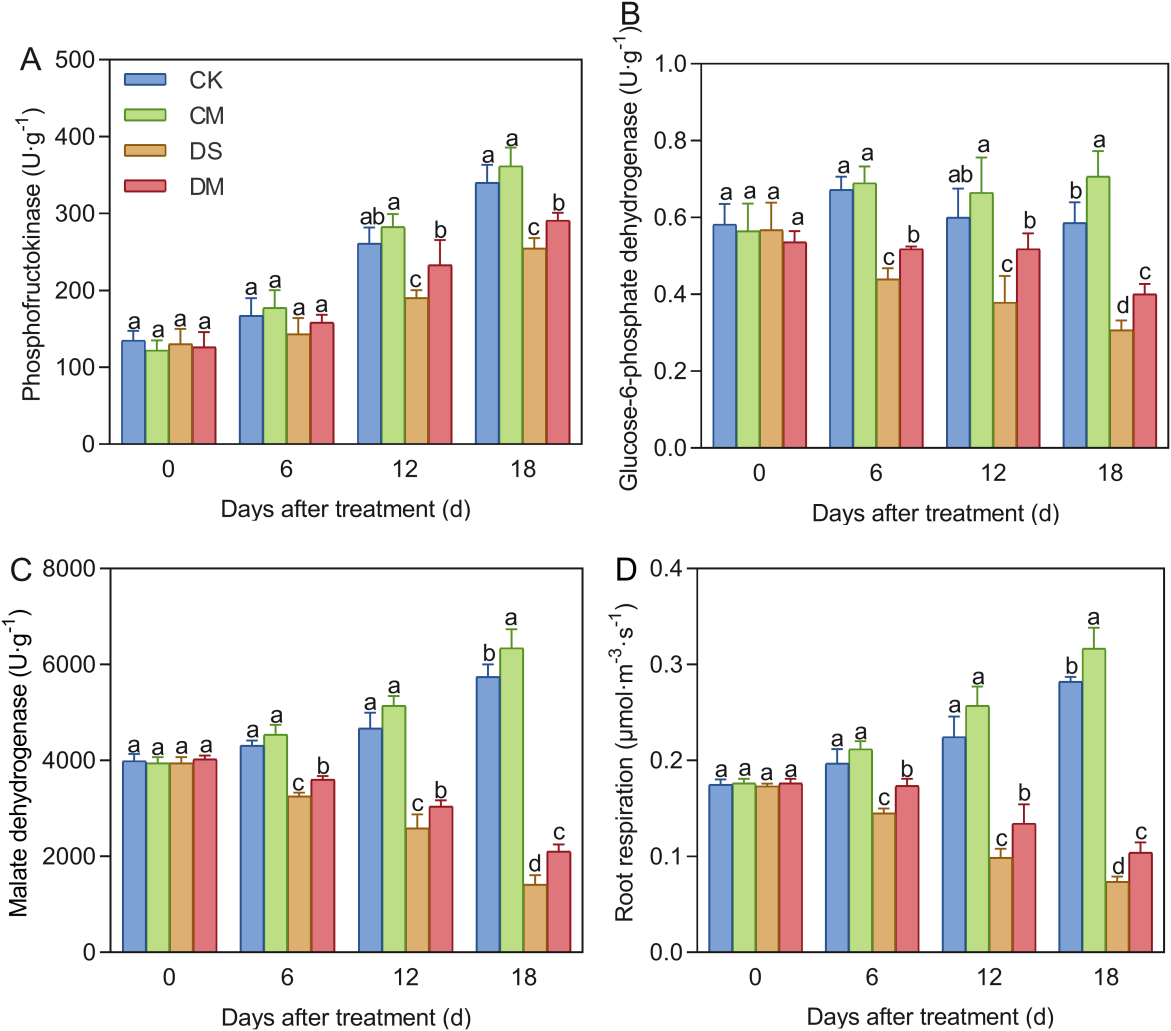
Effects of different treatments on root metabolism. **(A)** Phosphofructokinase, **(B)** Glucose-6-phosphate dehydrogenase, **(C)** Malate dehydrogenase, and **(D)** Specific root length respiration rate. CK, well water; CM, well water and melatonin; DS, drought stress; DM, drought stress and melatonin. Different lowercase letters indicate significant differences among different treatments at the same time point (*p* < 0.05). Values are means ± SE (n=3).

Melatonin significantly enhanced the activities of root respiratory metabolic enzymes and the root respiration rate in cotton seedlings under drought-stress treatment (**Figure 9**). Compared with the control, after 18 days of drought stress treatment, the activities of phosphofructokinase, glucose-6-phosphate dehydrogenase, malate dehydrogenase, and the specific root length respiration rate decreased by 25.16%, 47.50%, 75.37%, and 73.87%, respectively (*p* < 0.05). However, exogenous application of melatonin could significantly improve the normal level of root metabolism and mitigate the adverse effects of drought treatment. Specifically, compared with the well water treatment, the activities of glucose-6-phosphate dehydrogenase, malate dehydrogenase, and the specific root length respiration rate in the well water and melatonin treatment increased significantly by 20.73%, 10.45%, and 12.38%, respectively (*p* < 0.05); Meanwhile, compared with the drought stress treatment, the activities of phosphofructokinase, glucose-6-phosphate dehydrogenase, malate dehydrogenase, and the specific root length respiration rate of the drought stress and melatonin treatment increased significantly by 14.29%, 30.23%, 48.47%, and 41.06%, respectively (*p* < 0.05).

### 3.7 Correlation analysis

**Figure 10:**
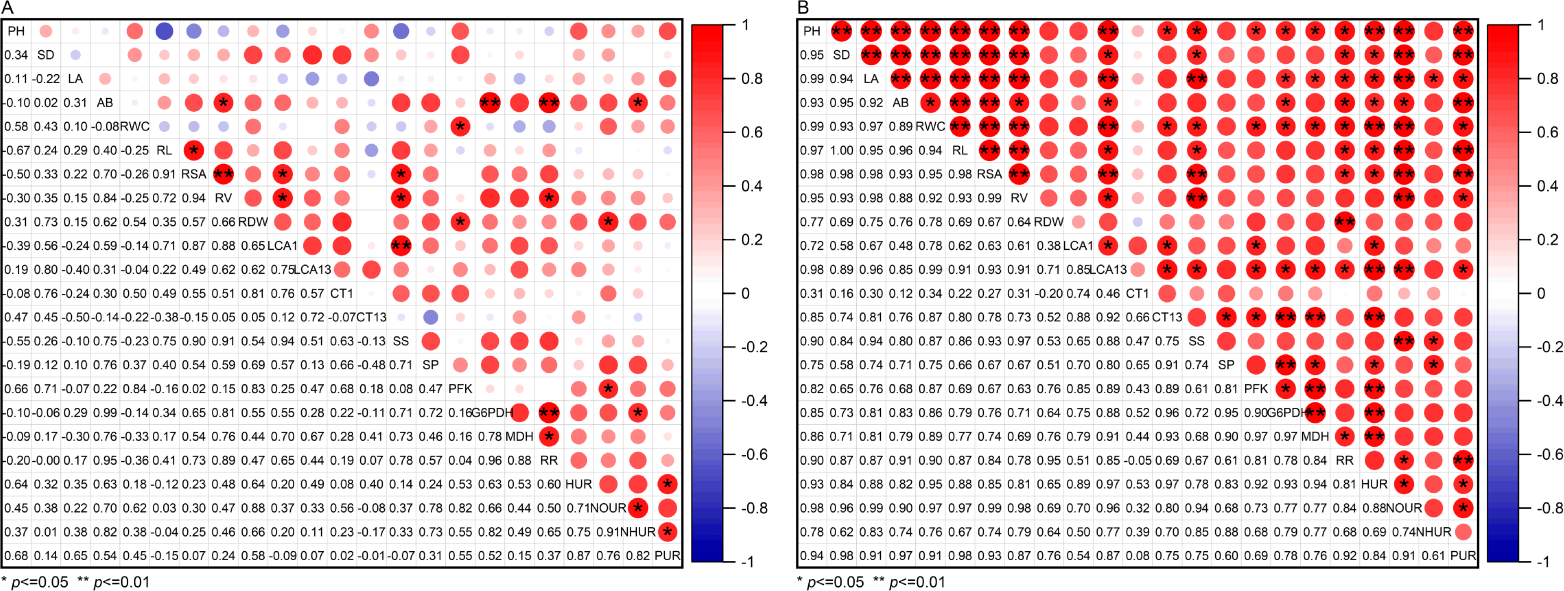
Correlation analysis of traits of cotton seedlings under well water **(A)** and drought stress **(B)** treatments with the application of melatonin. PH, Plant height; SD, Stem diameter; LA, Leaf area; AB, Above-ground biomass; RWC, Leaf relative water content; RL, Total root length; RSA, Total root surface area; RV, Total root volume; RDW, Root dry weight; LCA1, Living cortical area at 1 cm from the root tip; LCA13, Living cortical area at 13 cm from the root tip; CT1, Cortical thickness at 1 cm from the root tip; CT13, Cortical thickness at 13 cm from the root tip; SS, Soluble sugar content; SP, Soluble protein content; PFK, Phosphofructokinase; G6PDH, Glucose-6-phosphate dehydrogenase; MDH, Malate dehydrogenase; RR, Specific root length respiration rate; HUR, Specific root length Water uptake rate; NOUR, Specific root length Nitrate uptake rate; NHUR, Specific root length Ammonium uptake rate; PUR, Specific root length Phosphate uptake rate.

Correlation analysis revealed distinct relationships under different treatments (**Figure 10**). Under normal conditions, LCA of roots was significantly positively correlated with soluble sugars, This indicates that there was a significant positive correlation between root cortex cells and root osmotic adjustment under normal environmental conditions (**Figure 10A**).

As shown in **Figure 10B**, under drought stress, melatonin application strengthened these relationships: LCA correlated positively with soluble sugars, while cortical thickness aligned with soluble protein levels. Furthermore, LCA exhibited significant positive correlations with respiratory metabolism markers—phosphofructokinase, glucose-6-phosphate dehydrogenase, malate dehydrogenase, and specific root length respiration rate—as well as water, nitrate, and phosphate specific root length uptake rate. These cortical traits also positively correlated with root morphological parameters (total root length, total root surface area, and total root volume) and above-ground growth metrics (plant height, stem diameter, leaf area and above-ground biomass). In conclusion, exogenous melatonin promotes root growth by increasing LCA of roots under drought stress, facilitating osmotic substance accumulation, enhancing respiratory metabolism, and improving the root uptake rate, ultimately alleviating the damage caused by drought stress.

## 4 Discussion

### 4.1 Exogenous melatonin reduces the decline in root cortical cell activity under drought stress

Drought stress triggers programmed cell death in cortical cells, leading to root cortex damage. Studies have shown that drought stress alters the cortical structure of plant roots. In dicotyledonous plants such as grapevine, mechanical damage under drought leads to the formation of cavities in the fine root cortex, reducing LCA (Cuneo et al., 2016). In monocotyledons like wheat and barley, drought accelerates cortical cell sloughing through cortical cell death, resulting in a thinner cortex and a redistribution of remaining nutrients to rapidly growing root tissues (Liljeroth, 1995; Schneider et al., 2017). In this study, cotton, as a typical dicotyledonous plant, exhibited a progressive reduction in both LCA and cortex thickness with prolonged drought stress (**Figures 6**, **7**). This suggests that cortical shrinkage is a strategy plants use to reduce metabolic costs and adapt to drought stress, although it compromises root metabolism and active water uptake efficiency.

Melatonin protects root cortical cells. Exogenous application of melatonin has been confirmed to enhance drought resistance in plants (Dubbels et al., 1995; Guo et al., 2023). Our findings indicate that applying exogenous melatonin under drought stress alleviates root growth inhibition in cotton, significantly increasing total root length, total root surface area, total root volume, and root dry weight (**Figure 4**). However, under severe abiotic stress, maintaining a certain level of LCA is essential for root growth (Du et al., 2025). This study is the first to reveal that exogenous melatonin can reduce cortical tissue damage in cotton roots under drought conditions, significantly increasing LCA and cortical thickness at various distances from the root tip, thereby enhancing drought tolerance (**Figure 7**). This protective mechanism aligns with findings in sunflower under salt stress, confirming that regional accumulation of melatonin in root cortical cells regulates root growth and stress adaptation (Mukherjee et al., 2014).

The strategy by which plants enhance drought resistance through cortical tissue, as revealed in this study, differs markedly from the root cortical senescence mechanism reported in cotton by Guo et al. (2024b). Guo et al. demonstrated that cotton reduces root metabolic costs by accelerating cortical senescence or formation of cortical aerenchyma—to achieve “minimal metabolic consumption” (Guo et al., 2024b) (Schneider et al., 2017). For example, under water-limited conditions, tropical maize genotypes with high root cortical aerenchyma formation show significantly higher yields than those with low cortical aerenchyma formation (Chimungu et al., 2015b). In contrast, this study focuses on maintaining cortical cell activity through melatonin application, resulting in a 25.72–59.00% increase LCA (**Figure 7**), while simultaneously enhancing root metabolic activity and the efficiency of water and nutrient uptake (**Figures 5**, **9**), thereby achieving “maximum metabolic gain.” Moreover, the core distinction between these two strategies lies in their signaling regulation. Cortical senescence is typically governed by plant hormones such as ethylene, whose signaling pathways often lead to programmed cell death (Schneider et al., 2018). In contrast, melatonin helps plants reduce oxidative damage and delay programmed cell death by activating antioxidant and osmotic regulatory defense mechanisms, thus preserving cellular function (Hassan et al., 2022; Jiang et al., 2024).

This experiment focused on the lateral roots of cotton, a plant with a taproot system, primarily because lateral roots exhibit more active physiological processes and contribute a greater proportion to overall root biomass. To ensure developmental consistency and minimize experimental error, strict standardization of root sampling locations was implemented (Sidhu et al., 2024). Specifically, lateral roots approximately 20 cm in length and located 1 cm from the base of the main root were selected. Root segments measuring 0.5 cm in length were then excised at 1 cm and 13 cm from the lateral root tip (**Figure 1**). This highly uniform sampling strategy aimed to ensure that the collected samples were consistent in physiological status and structural characteristics, thereby enhancing the accuracy and reliability of the experimental data.

### 4.2 Exogenous melatonin modulates osmotic adjustment and respiratory metabolism to alleviate drought stress injury in plants

Under abiotic stress, plant cellular osmotic balance is disrupted. To maintain turgor pressure and enhance environmental adaptability, plants increase the accumulation of osmotic adjustment substances (Yancey, 2005). Exogenous melatonin can modulate related gene expression to promote the synthesis of these substances. For instance, melatonin treatment has been shown to upregulate genes involved in soluble sugar biosynthesis in early-maturing peach fruits, thereby increasing their content (Zhou et al., 2023). In this study, we found that after 6 days of drought stress, the contents of soluble sugars and soluble proteins in cotton roots were significantly higher than those in the control group, and exogenous melatonin significantly enhanced their accumulation (**Figure 8**). By day 18 of drought stress, the soluble protein content in the drought treatment was lower than that in the control, possibly due to the severity of stress at that stage (**Figure 8**). These results indicate that exogenous melatonin plays a crucial role in stress resistance by increasing the accumulation of osmotic adjustment substances under drought conditions.

Drought stress significantly affects the respiratory metabolic pathways in plant roots. In cotton, drought reduces the activities of key enzymes such as phosphofructokinase, glucose-6-phosphate dehydrogenase, and malate dehydrogenase, thereby impairing the plant’s energy supply (Guo et al., 2024b). Additionally, as drought intensifies and persists, the respiration rate of roots in many plant species declines (Fathi and Barari, 2016). In our study, the activities of respiratory enzymes and the respiration rate per unit root length in cotton roots were significantly reduced after 12 days of drought stress (**Figure 9**), indicating a strong inhibitory effect of drought on root metabolism. A drastic reduction in root metabolism can restrict plant growth and yield, and may even lead to plant death (Du et al., 2025). However, exogenous melatonin can promote the growth and development of cotton roots and enhance root vitality under drought stress, potentially by activating respiratory enzymes (Zhu et al., 2024). This study is the first to demonstrate that exogenous melatonin significantly increases the activities of phosphofructokinase, glucose-6-phosphate dehydrogenase, and malate dehydrogenase in cotton roots under drought stress, while also enhancing the respiration rate per unit root length (**Figure 9**). These findings suggest that melatonin may regulate respiratory metabolism by activating key enzymes, thereby supplying more energy and enhancing drought tolerance in cotton. This is consistent with the findings of Turk and Genisel, who reported that melatonin increased mitochondrial respiration and energy production in maize under cold stress (Turk and Genisel, 2020). Phosphofructokinase, a key rate-limiting enzyme in glycolysis, may exhibit increased activity with prolonged drought exposure (**Figure 9**), thereby accelerating carbohydrate breakdown and providing more intermediate metabolites and energy for root function (Dwivedi et al., 2023).

The relationship between root respiratory metabolism and cortical activity as a source of energy is central to plant environmental adaptation. LCA is closely linked to respiratory intensity; thus, regulating the formation of root cortical aerenchyma or promoting cortical senescence helps reduce respiratory consumption (Lynch et al., 2014; Chimungu et al., 2015a; Wojciechowska et al., 2021). Using the SimRoot model, simulations under phosphorus stress showed that after 40 days of growth, the formation of root cortical aerenchyma reduced root respiration and accelerated growth in common bean and maize (Postma and Lynch, 2011). Therefore, cortical cell activity is positively correlated with root respiratory metabolism. This aligns with our findings: in cotton seedlings, LCA is positively correlated with respiratory enzyme activity and respiration rate per unit root length (**Figure 10**). Melatonin, as a mitochondrial protector, scavenges reactive oxygen species and reactive nitrogen species generated by the respiratory chain, thereby maintaining the integrity of electron transport and ensuring continuous ATP synthesis (Laxa et al., 2019). Under drought stress, although the application of melatonin increases LCA—leading to elevated basal metabolic consumption—it also regulates various physiological and biochemical responses to achieve a net positive energy balance. This ultimately enhances drought tolerance in cotton.

### 4.3 Exogenous melatonin improves water and nutrient uptake rates under drought stress

Roots are the primary organs responsible for water and nutrient uptake in plants, and their absorptive capacity directly influences plant growth, development, and stress resilience (Fitter et al., 2002). Under adverse environmental conditions, the decline in cortical cell activity and weakened respiratory metabolism in roots reduces the energy available for active ion transport. This leads to decreased uptake rates of essential nutrients such as nitrate, ammonium, and phosphate ions. Meanwhile, the synthesis and functioning of aquaporins and nutrient transporters on cell membranes are also impaired, further hindering water and nutrient uptake and transportation (Li et al., 2020; Hao et al., 2023). Melatonin plays a positive regulatory role by enhancing the plant defense system, mitigating oxidative damage, and maintaining normal root functionality, thereby minimizing the adverse effects on root uptake capacity (Sun et al., 2022). Previous studies have demonstrated that during drought stress in maize, PEG-induced water deficiency significantly decreases root hydraulic conductivity. However, the application of melatonin significantly up-regulates the transcriptional levels of aquaporin genes, increases root hydraulic conductivity, improves root water uptake and transport efficiency, and thereby enhances maize’s drought tolerance (Qiao et al., 2020). In apples, drought stress significantly reduces the root absorption flux of nitrogen, phosphorus, and potassium. Melatonin facilitates nitrogen uptake by modulating the expression and activity of nitrogen-related transporters and promotes phosphorus uptake by accelerating the secretion of acid phosphatases from the roots (Liang et al., 2018). In this study, we found that exogenous melatonin significantly enhanced the uptake rates of water, nitrate, ammonium, and phosphate in cotton roots under drought stress (**Figure 5**). Thus, melatonin improves plant water and nutrient use efficiency under drought by regulating nutrient absorption, thereby supporting normal physiological function of the roots.

Exogenous melatonin increases LCA of roots and promotes the accumulation of osmotic regulatory substances, thereby improving water and nutrient uptake under drought conditions. On the one hand, an increased LCA expands the contact area between roots and soil and increases root hair density, leading to enhanced water uptake (Tanaka et al., 2014). The structural integrity of the root cortex strengthens intercellular connectivity and material transport pathways, promoting the uptake, translocation, and storage of water and nutrients from the soil, which improves plant survival under stress conditions (Cuneo et al., 2016). On the other hand, drought-induced reactive oxygen species trigger membrane lipid peroxidation, which reduces membrane fluidity and increases permeability. This impairs the function of membrane transport proteins, causes leakage of intracellular substances, and decreases the capacity for water and nutrient uptake, even leading to cell death (Gill and Tuteja, 2010). Melatonin activates the antioxidant defense and osmotic adjustment systems, alleviates oxidative damage, and lowers cellular water potential, enabling water to move from the soil into root cells along the water potential gradient (Ozturk et al., 2021). Osmotic adjustment substances protect biofilms, maintain the integrity of the plasma membrane of root cortex cells, and ensure normal water and nutrient uptake and transport (Singh et al., 1972); and maintain the integrity of mitochondrial membranes, ensuring ATP synthesis and providing energy for water and nutrient uptake (Griffiths et al., 2021).

Enhanced water and nutrient uptake directly supplies plants with adequate water to meet their physiological needs under drought conditions. Rapid water absorption ensures that leaf photosynthesis and transpiration proceed normally, providing the energy and raw materials needed for the synthesis of organic compounds (von Caemmerer and Baker, 2006; Renger, 2007). Improved nutrient uptake delivers sufficient mineral elements for the synthesis of physiological compounds that enhance drought tolerance (Ayyaz et al., 2024). Therefore, strengthening root absorptive capacity significantly improves drought resistance in cotton. These physiological processes interact in a complex and dynamic network, collectively enhancing the plant’s overall drought adaptability. In this experiment, PEG-induced hydroponics was selected to determine water and nutrient uptake rates. However, PEG can only alter osmotic potential and cannot simulate soil mechanical resistance, matrix potential, or the microbial interactions typical of field drought (Verslues et al., 2018) (Backer et al., 2018). Therefore, field validation is still required for this experiment in the future.

## 5 Conclusions

**Figure 11:**
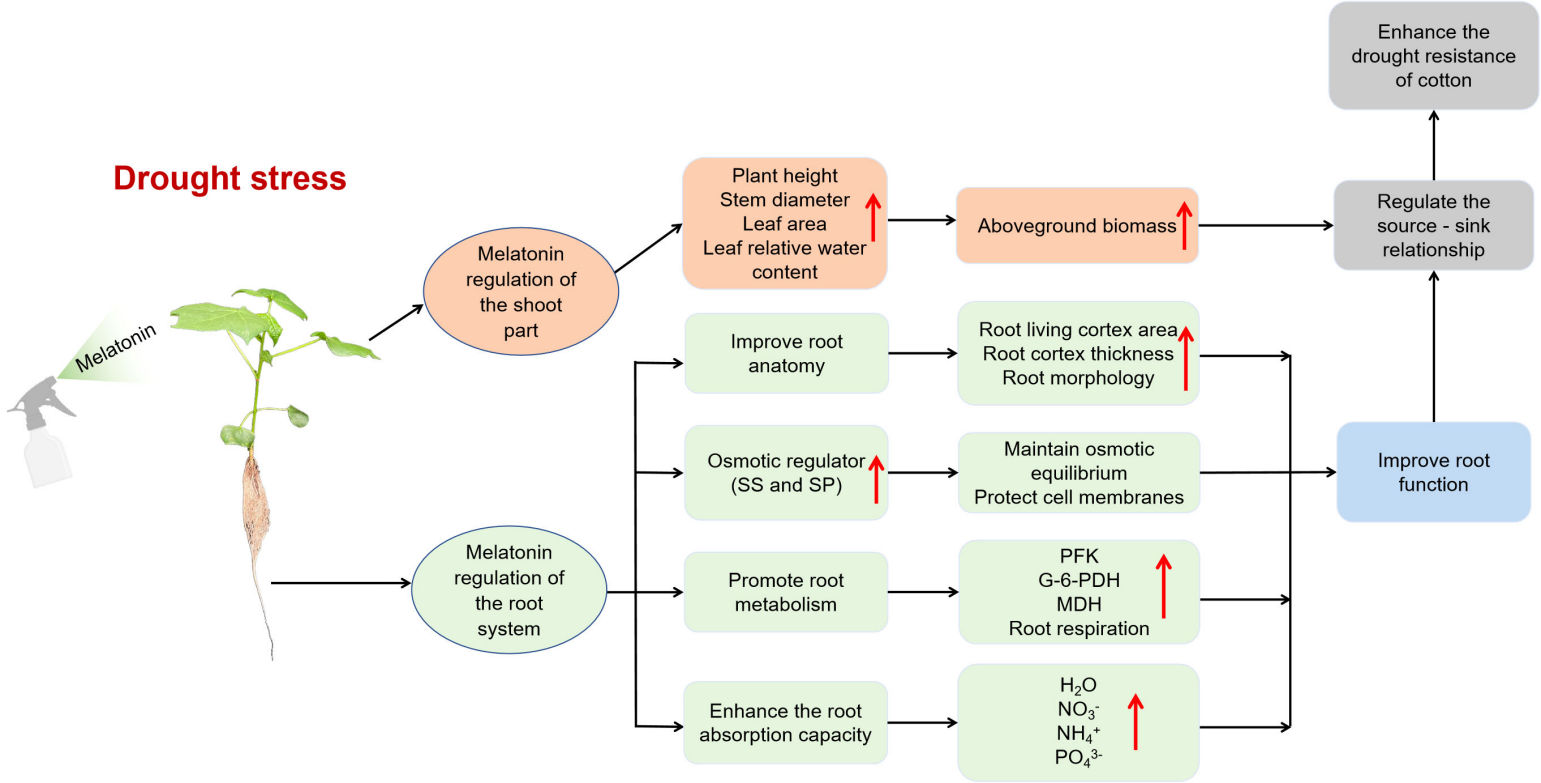
Schematic diagram of the mechanism by which exogenous melatonin enhances the drought resistance of cotton under drought stress. SS, Soluble sugar content; SP, Soluble protein content; PFK, Phosphofructokinase; G-6-PDH, Glucose-6-phosphate dehydrogenase; MDH, Malate dehydrogenas.

This study fills a critical gap in the literature regarding the effects of melatonin on root cortical cells under drought stress and demonstrates its role in enhancing drought tolerance in cotton. Drought stress significantly reduces LCA of roots, severely impairing root function. Our findings highlight that exogenous melatonin promotes cotton root growth by increasing cortical cell activity, enhancing the accumulation of osmotic regulators, boosting respiratory metabolism, and improving the efficiency of water and nutrient uptake. These physiological improvements help maintain leaf water balance, enhance above-ground biomass accumulation, and optimize source–sink relationships, thereby alleviating the growth-inhibitory effects of drought and ultimately improving drought resistance (**Figure 11**). Therefore, increasing LCA through melatonin application under drought stress may serve as a sustainable strategy to enhance the plant’s capacity to access deep-soil resources and mitigate drought-induced damage in crops.

## Data Availability

The raw data supporting the conclusions of this article will be made available by the authors, without undue reservation.
