# Supplementary figures and images for: Exogenous melatonin alleviates drought stress in cotton by enhancing root cortical activity and metabolic adaptation
Source: Front Plant Sci. 2025 Jul 2;16:1625757. doi: 10.3389/fpls.2025.1625757 (PMC12264641; doi:10.3389/fpls.2025.1625757)

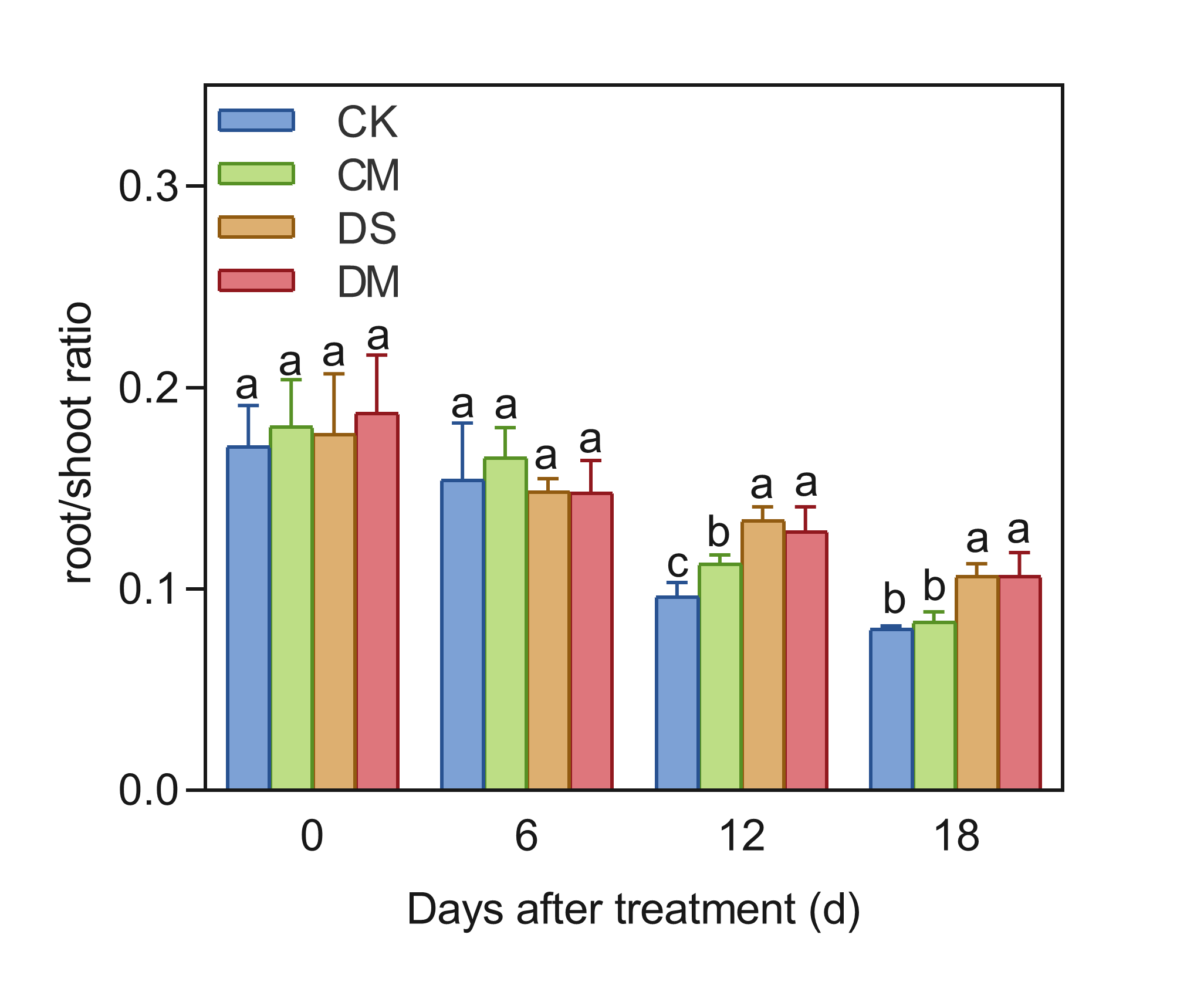

Supplement: Supplementary file 1 [file Image1.tif]
